# Health-Related Quality of Life in Turner Syndrome and the Influence of Growth Hormone Therapy: A 20-Year Follow-Up

**DOI:** 10.1210/jc.2019-00340

**Published:** 2019-04-22

**Authors:** Emily Krantz, Kerstin Landin-Wilhelmsen, Penelope Trimpou, Inger Bryman, Ulla Wide

**Affiliations:** 1 Clinic of Internal Medicine, Södra Älvsborgs Hospital, Borås, Sweden; 2 Department of Internal Medicine and Clinical Nutrition, Institute of Medicine, Sahlgrenska Academy, University of Gothenburg, Gothenburg, Sweden; 3 Section of Endocrinology, Sahlgrenska University Hospital, Gothenburg, Sweden; 4 Department of Reproductive Medicine, Sahlgrenska University Hospital, Gothenburg, Sweden; 5 Department of Obstetrics and Gynecology, Institute of Clinical Sciences, Sahlgrenska Academy, University of Gothenburg, Gothenburg, Sweden; 6 Department of Behavioral and Community Dentistry, Institute of Odontology, Sahlgrenska Academy, University of Gothenburg, Gothenburg, Sweden

## Abstract

**Context:**

The factors that affect the health-related quality of life (HRQoL) of women with Turner syndrome (TS) are controversial.

**Objective:**

The aim was to describe the HRQoL of women with TS with a focus on how given GH treatment and comorbidity influence HRQoL in adulthood and to compare HRQoL of women with TS with that of women in the general population.

**Design:**

Longitudinal cohort study, up to 20 years.

**Setting:**

The Turner Center at the Section for Endocrinology and Department of Reproductive Medicine at Sahlgrenska University Hospital, Gothenburg, Sweden.

**Participants:**

Women with TS (n = 200), age range 16 to 78 years, were included consecutively and monitored every fifth year between 1995 and 2018. Women from the World Health Organization MONItoring of trends and determinants for CArdiovascular disease project were used as reference populations.

**Interventions and Main Outcome Measures:**

HRQoL was measured using the Psychological General Well-Being index and the Nottingham Health Profile. Associations with somatic variables were assessed using longitudinal linear regression models.

**Results:**

HRQoL was not associated with GH treatment in TS in spite of a mean 5.7 cm taller height. HRQoL was only associated with height *per se* in one of 13 subscales (*P* < 0.01). HRQoL was negatively affected by higher age, higher age at diagnosis, and hearing impairment in TS. Women with TS reported a similar HRQoL to the reference population.

**Conclusions:**

No association between previous GH treatment and HRQoL was found during the up to 20 years of follow-up in women with TS. HRQoL of women with TS and the reference population was similar.

## Teaser

In this 20-year follow-up study of HRQoL of women with Turner syndrome, no association between HRQoL and previous GH treatment was found, calling the relevance of GH treatment into question.

Turner syndrome (TS) is characterized by a total or partial loss of one of two X chromosomes, affecting ∼1 in 2500 female births. It is associated with physical features (or stigmata) such as short stature, cubitus valgus, webbing of the neck, lymphedema, and pubertal delay (1, 2). Serious clinical manifestations of TS require regular attention in adulthood like ovarian insufficiency, cardiovascular anomalies and risk for aortic dissection, osteoporosis, hypothyroidism, hearing loss, neurodevelopmental challenges, and social anxiety (3–6). It is important to measure the extent to which the different manifestations of TS, and subsequent treatments, affect health-related quality of life (HRQoL) (7).

The definition of HRQoL applied in this study is “the functional effect of an illness or disorder and its consequent therapy upon a patient, as perceived by the patient” (8). It is, therefore, subjective, and a multidimensional approach has been taken to include aspects of physical and occupational function, psychological state, and social interaction. A recent meta-analysis of HRQoL of women with TS concluded that their HRQoL seems to be compromised, but the factors that affect it in adulthood remain controversial (9). Furthermore, results were inconsistent regarding whether growth-promoting therapy administered in childhood is of benefit from a HRQoL perspective for these patients.

Children with short stature have been offered GH in Sweden since 1986, and GH treatment in girls with TS began in 1988. TS is not associated with GH deficiency; the sole objective of the treatment is to increase final height, and it has been shown that it does so effectively (10). However, only one study based on a randomized, placebo-controlled trial of GH treatment in girls with TS has been published that included HRQoL outcomes (11). Most other studies that draw conclusions on GH treatment’s influence on HRQoL in TS are either very small (12) or do so in comparison with women in the population and do not compare the GH-treated women to the untreated women (13–16). There is a knowledge gap regarding the long-term effects of GH on HRQoL in adulthood in TS (17).

The aim of this long-term follow-up of a previous study (18) was to describe HRQoL of adult women with TS with a focus on how GH treatment and comorbidity influence HRQoL during adulthood and to compare the HRQoL of women with TS, with that of women in the general population. The hypotheses were that the younger generation of women with TS who received GH would have a better HRQoL than the women with TS who did not receive modern treatment and that the HRQoL of the women with TS would be lower compared with the women in the population.

## Materials and Methods

### Study setting

This longitudinal cohort study was conducted at the Turner Center at the Section for Endocrinology and the Department of Reproductive Medicine at Sahlgrenska University Hospital, Gothenburg, Sweden, between 1995 and 2018. Women with TS were monitored according to the Swedish and international clinical practice guidelines for TS (6, 19). All of the patients were examined by the same internal medicine specialist/endocrinologist (K. L.-W.) and gynecologist (I. B.) during the entire follow-up time.

### Subjects

**Figure 1.**
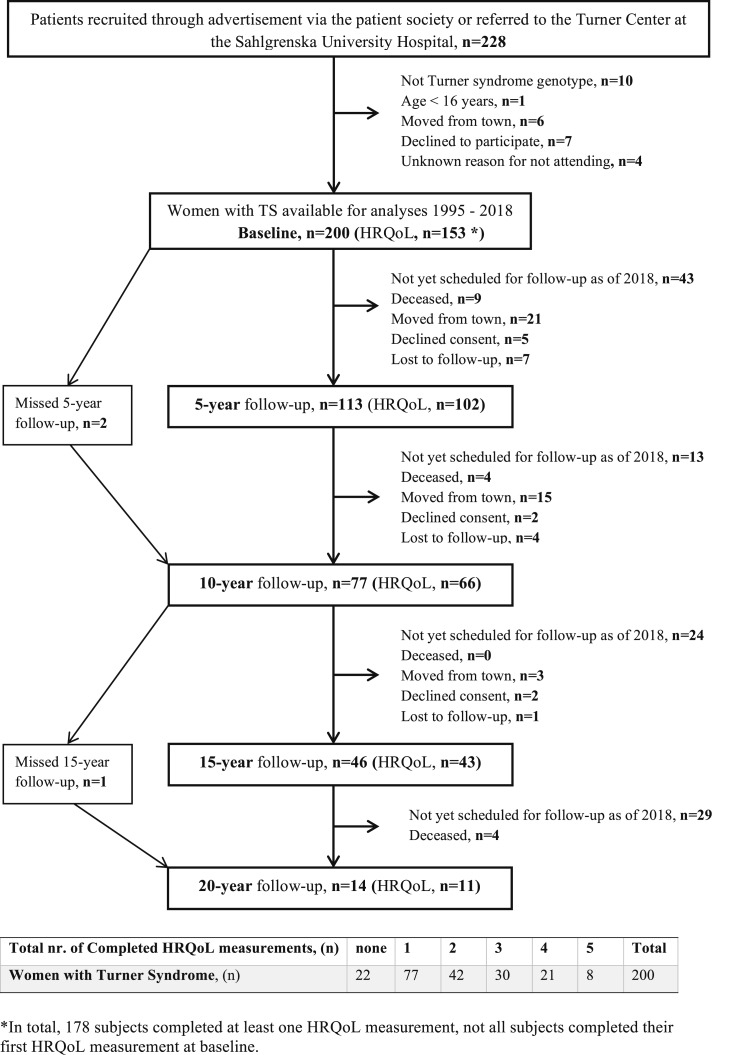
Flow chart and frequency table for the consecutive inclusion of women with TS and follow-up visits.

Patients with suspected or diagnosed TS were recruited through an advertisement in the Turner patient magazine, by referral from the hospitals, or transferred from the pediatric clinics in the county of Västra Götaland with 1.5 million inhabitants. Inclusion was consecutive, and criteria were phenotypic subjects with TS and a partial or complete absence of an X chromosome in at least 5% of leukocytes or buccal cells. The study sample is presented from inclusion through 20 years of follow-up in Fig. 1.

All women ≥18 years of age (n = 200) were asked to complete two HRQoL questionnaires (specified below) every fifth year. HRQoL questionnaires were completed at least once by 178 women, but not necessarily at baseline (89% participation rate in HRQoL measurement). The 22 women who did not complete any HRQoL questionnaires actively declined consent (n = 5), were not offered HRQoL questionnaires because they were under the age of 18 at baseline (n = 4), or were not offered HRQoL questionnaires because of an administrative error or did not turn in the questionnaires at the end of their visit (n = 13).

Of the 178 women with TS who participated in the HRQoL measurements, 101 women completed at least one HRQoL follow-up (*i.e.*, have completed the questionnaires at least twice). Of the 77 women who only completed the one measurement: 35 had not yet been scheduled for a follow-up as of August 2018, 7 were deceased, 19 had moved to another city, 9 declined consent or were not offered HRQoL questionnaires because of an administrative error, and 7 were lost to follow-up. Details of the number of HRQoL follow-ups the women with TS completed can be found in Fig. 1.

### Reference population

A randomly selected population-based sample of women served as a reference population from Gothenburg, Sweden, in the third population sample in the World Health Organization (WHO) MONItoring of trends and determinants for CArdiovascular disease (MONICA-GOT) (20). In 1995, 2612 individuals (aged 25 to 64; 50% women) were recruited from the city census, which is kept up to date within a maximum of 14 days (n = 872 women participated). Of these, every fourth woman in the age range 25 to 44 years and all of the women in the age range 45 to 64 years, in total 558 subjects, underwent extended hormonal sampling. Those who were alive in 2007 were invited for re-evaluation and assessment of HRQoL. Details regarding inclusion and dropout can be read elsewhere (20, 21). In total, 317 women were examined at a medical clinic and completed the HRQoL questionnaires (64% participation rate; average age 64 ± 9 years, minimum to maximum 39 to 78 years), and were used as a reference population.

### HRQoL measurement instruments

Each subject completed the validated Swedish language versions of the Psychological General Well-Being index (PGWB) and the Nottingham Health Profile (NHP).

The PGWB was designed to measure personal affective or emotional states reflecting a sense of well-being or distress intended for use in community surveys (22). The PGWB includes 22 items, with a six-grade Likert style response format in which a high score represents a better HRQoL. The scores are summarized into an overall well-being score (PGWB total score, range 22 to 132) and also divided into six subscales: Anxiety (range 5 to 30), Depressed Mood (range 3 to 18), Positive Well-being (range 4 to 24), Self-control (range 3 to 18), General Health (range 3 to 18), and Vitality (range 4 to 24). PGWB has been used in clinical trials and has performed well in both population-based and mental health samples (23).

The NHP measures aspects of subjective health (24), and, in this study, only part I was used. Thirty-eight statements (response format is yes or no) cover six dimensions concerning distress or limitations of activity: Physical Mobility, Pain, Sleep, Energy, Social Isolation, and Emotional Reactions. Dimension scores range from 0 to 100, and each statement is weighted according to the level of severity. The higher the score, the greater the limitations/distress (*i.e.*, the lower HRQoL). The NHP is useful because of its breadth and simplicity and is a suitable instrument for use in clinical practice and in populations in which there are likely to be people with disabilities (25).

### Anthropometry and social variables

Physical examinations and medical history were taken and recorded every fifth year including age, height, body weight, all medications, comorbidities, use of hearing aid, smoking status, marital status (married/cohabiting or not), children in family (own or adopted), occupation (employed/student), at sick leave or disability pension, and degree of physical activity (sedentary, moderate, or regular), by the same specialists in all patients. The number of external TS stigmata, genotype, previous GH therapy [mean dose 0.05 mg/kg/d from 3 to 14 years of age (19)], education level (completed secondary school), and the presence of a cardiovascular malformation by echocardiography (bicuspid aortic valve and coarctation of the aorta) were all recorded at baseline. Similar examinations and collection of medical history were carried out on the subjects in the reference population by K.L.-W. and P.T. (18, 21). The chromosome status in the TS women was based on both karyotyping and fluorescence *in situ* hybridization (26).

### Ethical considerations

This study was approved by the Regional Ethical Review Board in Gothenburg (Dnr 456-94, Dnr S572-99, Dnr 242-02, Dnr 088-06, and Dnr T282-11), and all participants gave their informed consent.

### Statistics

Means and SD were calculated using conventional methods. Group comparisons of continuous variables were made with Student *t* test for the somatic variables. Fisher exact test was used to compare groups for discreet data. ANOVA was used to compare HRQoL outcomes when an adjustment for age was necessary [univariate ANOVA (UNIANOVA)]. Due to a limited amount of data, linear regression models were applied when analyzing the longitudinal data. In some of the analyses, it was possible to apply a random effects model (random intercept on an individual level), but it was concluded that the results only changed marginally compared with the results from the ordinary linear regression. Therefore, for the sake of simplicity when presenting the data, only ordinary linear regression results are shown and interpreted. Because of the complexity of the dataset, 99% CIs were calculated, and *P* < 0.01 (two-sided test) was considered statistically significant to reduce the risk of type I error. All statistical analyses were calculated using the Statistical Package for the Social Sciences (SPSS v. 24; SPSS Inc., Chicago, IL) software.

## Results

### Background data and medication

Background data at baseline and at each follow-up for women with TS and for the reference population are shown in Table 1. The average age of the women with TS at baseline was 28 ± 11 years (minimum to maximum 16 to 71). Eighteen women with TS died during the entire follow up period (average age at death was 50 years; minimum to maximum 25 to 75) (Fig. 1).

**Table 1.** Anthropometric, Treatment, and Social Background Data for Women With TS at Baseline Starting in 1995 and 5–20 y Follow-Up and for the Reference Population of Women From WHO-MONICA in 1995 and at 13 y Follow-Up in 2008

	TS Baseline (n = 200)	TS 5-y Follow-Up (n = 113)	TS 10-y Follow-Up (n = 77)	TS 15-y Follow-Up (n = 47)	TS 20-y Follow-Up (n = 14)	Reference Population 1995 (n = 400)	Reference Population Re-examined (n = 317)
Age, y, mean (SD); minimum–maximum	28.2 (11.2); 16–71^*a*^	35.4 (11.1); 22–66	41.8 (11.2); 27–67	50.2 (11.8); 34–74	51.8 (10.6); 40–78	35.4 (5.7); 25–45	63.7 (9.0); 39–78
Height, cm, mean (SD)	153.5 (6.8)^*a*^	152.1 (6.6)	151.7 (6.1)	150.7 (6.8)	149.6 (6.9)	166.3 (6.6)	163.4 (6.3)
Body weight, kg, mean (SD)	59.2 (12.9)^*a*^	59.4 (13.4)	59.4 (12.4)	58.6 (12.6)	61.9 (13.2)	65.2 (10.2)	71.2 (14.0)
Body mass index, kg/m^2^, mean (SD)	25.0 (4.6)^*a*^	25.5 (5.2)	25.7 (4.9)	25.5 (4.9)	27.7 (6.2)	23.6 (3.5)	26.7 (5.0)
Waist-hip-ratio, mean (SD)	0.81 (0.08)^*a*^	0.83 (0.09)	0.84 (0.07)	0.86 (0.08)	0.86 (0.06)	0.78 (0.05)	0.84 (0.08)
Treatment, n (%)							
GH treatment (previous)	125 (63)	59 (52)	35 (46)	15 (32)	7 (50)	n.a.	n.a.
Estrogen replacement (current)	159 (80)^*a*^	100 (88)	71 (92)	42 (89)	11 (78)	71 (18)	27 (8)
Antihypertensive agents	16 (8)^*a*^	16 (14)	18 (23)	19 (40)	7 (50)	14 (4)	94 (30)
Lipid-lowering agents	6 (3)	1 (0.8)	2 (3)	2 (4)	1 (7)	n.a.^*b*^	49 (16)
Levothyroxine supplementation	46 (24)^*a*^	36 (32)	33 (43)	21 (45)	9 (64)	8 (2)	37 (12)
Bone-specific agents	1 (0.5)	2 (2)	3 (4)	3 (6)	0 (0)	n.a.^*b*^	13 (4)
Diabetes therapy	2 (1)	1 (0.8)	1 (1)	2 (4)	0 (0)	3 (1)	7 (2)
Antidepressant and/or sedatives	10 (5)	16 (14)	13 (17)	7 (15)	3 (21)	24 (6)	72 (23)
Analgesic (regular use)	10 (5)	11 (9)	7 (9)	3 (6)	1 (7)	30 (8)	52 (17)
Hearing aid, n (%)	26 (13)	21 (19)	28 (36)	25 (53)	10 (71)	n.d.	39 (12)
Married/cohabiting, n (%)	58 (32)^*a*^	53 (47)	37 (48)	24 (51)	9 (64)	258 (65)	173 (55)
Women with children in family, own and adopted, n (%)	20 (9)	22 (20)	18 (23)	14 (30)	5 (35)	n.d.	264 (83)
Employed/student, n (%)	179 (90)	91 (81)	61 (79)	34 (72)	11 (79)	340 (85)	n.a.^*c*^
Sick leave/disability pension, n (%)	11 (6)	8 (7)	7 (9)	4 (8)	2 (14)	18 (5)	n.a.^*c*^
Completed secondary school during follow-up time, n (%)	156 (78)^*a*^					266 (70)	147 (46)
Physical exercise, n (%)							
Sedentary	39 (20)^*a*^	14 (12)	7 (9)	8 (17)	3 (21)	41 (15)	43 (14)
Moderate	77 (39)	44 (39)	45 (58)	30 (64)	11 (79)	179 (66)	197 (62)
Regular	59 (30)	33 (29)	17 (22)	3 (6)	0 (0)	50 (18)	77 (24)
Tobacco user, n (%)	14 (7)^*a*^	9 (8)	3 (4)	1 (2)	0 (0)	76 (28)	30 (10)
Heart malformation (bicuspid and/or coarctation of aorta), n (%)	53 (27)					n.d.	n.d.
Number of stigmata, mean (SD)	6.9 (3.7)					n.a.	n.a.
Age at diagnosis, mean (SD); minimum–maximum	10.4 (9.7); 0–58					n.a.	n.a.
Karyotype, n (%)						n.a.	n.a.
Monosomy X (45,X)	90 (45)						
Mosaic (45.X/46,XX)	32 (16)						
Mosaicism with “Triple X” (45,X/47,XXX or 45,X/46,XX/47,XXX)	4 (2)						
Mixed gonadal dysgenesis (45,X/46,XY or 45,X/46,XX/47,XY)	20 (10)						
Mosaic with iso-chromosome	33 (16)						
Mosaic with ring chromosome	8 (4)						
Deletion (Xp22.3)	11 (6)						
Translocation	2 (1)						

Abbreviations: n.a., not applicable; n.d., no data.

^a^ *P* < 0.01, TS at baseline compared with reference population 1995 (Student *t* test or Fisher exact test).

^b^ Lipid-lowering and bone-specific agents were not registered for use in 1995 in Sweden.

^c^ Majority of subjects retired at 65 y of age.

GH treatment in childhood was used by 63% among women with TS (Table 2). The GH-treated women were on average 17 years younger and 5.7 cm taller at baseline than those who had not received GH treatment. Comorbidity was similarly distributed between the GH-treated and nontreated women with TS.

**Table 2.** Anthropometric, Treatment, and Social Background Data at Baseline and 5–20 y Follow-Up for Women With TS, Without and With Previous GH Treatment (GH−/GH+)

	TS Baseline		TS 5-y Follow-Up		TS 10-y Follow-Up		TS 15-y Follow-Up		TS 20-y Follow-Up	
	GH−, n = 75	GH+, n = 125	GH−, n = 54	GH+, n = 59	GH−, n = 42	GH+, n = 35	GH−, n = 31	GH+, n = 15	GH−, n = 7	GH+, n = 7
Age, y, mean (SD); minimum–maximum	38.9 (11.5); 19–71^*a*^	21.8 (3.4); 16–35	44.0 (10.0); 30–66^*a*^	27.5 (3.4); 22–39	59.8 (9.2); 38–67^*a*^	32.3 (2.6); 27–37	55.8 (10.2); 43–74^*a*^	38.3 (2.2); 34–42	59.7 (9.6); 49–78^*a*^	43.9 (2.2); 40–47
Height, cm, mean (SD)	149.9 (7.2)^*a*^	155.6 (5.7)	149.3 (6.2)^*a*^	154.5 (6.1)	149.8 (6.3)^*a*^	153.9 (5.2)	149.6 (6.9)	153.4 (5.7)	149.6 (8.7)	149.5 (5.2)
Body weight, kg, mean (SD)	57.8 (14.5)	60.1 (12.0)	57.7 (12.1)	61.0 (14.7)	59.1 (11.7)	59.7 (12.9)	58.3 (12.0)	59.3 (14.2)	68.8 (13.2)	54.8 (9.6)
Body mass index, kg/m^2^, mean (SD)	25.4 (5.3)	24.7 (4.2)	25.5 (5.1)	25.6 (5.3)	26.2 (4.8)	25.2 (5.1)	25.8 (4.8)	24.8 (5.2)	30.8 (6.1)	24.6 (4.9)
Waist-hip ratio, mean (SD)	0.83 (0.08)^*a*^	0.79 (0.07)	0.85 (0.09)	0.82 (0.09)	0.85 (0.07)	0.82 (0.06)	0.89 (0.07)	0.83 (0.08)	0.89 (0.07)	0.84 (0.05)
Treatment, n (%)										
Estrogen replacement (current)	56 (75)	103 (82)	46 (85)	54 (92)	39 (93)	32 (91)	27 (87)	14 (93)	5 (71)	6 (86)
Antihypertensive agents	15 (20)^*a*^	1 (1)	13 (24)^*a*^	3 (5)	13 (31)	5 (14)	14 (45)	5 (33)	5 (71)	2 (29)
Lipid-lowering agents	4 (5)	2 (2)	1 (2)	0 (0)	2 (5)	0 (0)	2 (6)	0 (0)	0 (0)	1 (14)
Levothyroxine supplementation	9 (12)^*a*^	39 (31)	11 (20)	25 (42)	17 (40)	16 (38)	14 (45)	7 (47)	5 (71)	4 (57)
Bone-specific agents	1 (1)	0 (0)	2 (4)	0 (0)	2 (5)	0 (0)	3 (10)	0 (0)	0 (0)	0 (0)
Diabetes therapy	1 (1)	1 (1)	1 (2)	0 (0)	1 (2)	0 (0)	2 (6)	0 (0)	0 (0)	0 (0)
Antidepressant and/or sedatives	5 (7)	5 (4)	11 (20)	5 (8)	9 (21)	4 (11)	6 (19)	1 (7)	2 (29)	1 (14)
Analgesic (regular use)	8 (11)^*a*^	2 (2)	9 (16)	2 (3)	5 (12)	2 (6)	3 (10)	0 (0)	1 (14)	0 (0)
Hearing aid, n (%)	18 (24)^*a*^	8 (6)	17 (31)^*a*^	4 (7)	19 (45)	9 (26)	19 (61)	6 (40)	5 (71)	5 (71)
Married/cohabiting, n (%)	34 (45)^*a*^	29 (23)	28 (52)	25 (42)	19 (45)	18 (51)	14 (45)	9 (60)	4 (57)	5 (71)
Women with children in family, own or adopted, n (%)	14 (19)^*a*^	3 (2)	17 (31)^*a*^	6 (10)	13 (31)	5 (14)	8 (26)	6 (40)	1 (14)	4 (57)
Employed/student, n (%)	63 (84)	116 (93)	43 (80)	48 (81)	31 (74)	29 (83)	21 (68)	13 (86)	5 (71)	6 (86)
Sick leave/disability pension, n (%)	7 (9)	4 (3)	6 (11)	2 (3)	5 (12)	2 (6)	4 (13)	0 (0)	1 (14)	1 (14)
Completed secondary school, n (%)	49 (65)^*a*^	107 (86)								
Physical exercise: n (%)										
Sedentary	19 (25)	20 (16)	9 (17)	5 (8)	5 (21)	2 (6)	7 (23)	1 (7)	3 (43)	0 (0)
Moderate	24 (32)	53 (42)	16 (36)	28 (47)	24 (9)	21 (60)	21 (68)	9 (60)	4 (57)	7 (100)
Regular	21 (28)	38 (30)	13 (24)	20 (34)	9 (21)	8 (23)	1 (3)	1 (7)	0 (0)	0 (0)
Tobacco user, n (%)	8 (11)	6 (5)	5 (9)	4 (7)	1 (2)	2 (6)	1 (3)	0 (0)	0 (0)	0 (0)
Number of stigmata, mean (SD)	7.5 (4.0)	6.3 (3.3)								
Age at diagnosis, mean (SD); (minimum**–**maximum)	16.3 (12.5); (0–58)^*a*^	6.9 (5.3); (0–16)								
Karyotype: n (%)										
Monosomy X (45,X)	33 (44)	57 (46)								
Mosaic (45,X/46,XX)	15 (20)	17 (14)								
Mosaicism with “Triple X”	1 (1.3)	3 (2)								
(45,X/47,XXX or 45,X/46,XX/47,XXX)										
Mixed gonadal dysgenesis	10 (13)	10 (8)								
(45,X/46,XY or 45,X/46,XX/47,XY)										
Mosaic with iso-chromosome	10 (13)	23 (18)								
Mosaic with ring chromosome	1 (1.3)	7 (6)								
Deletion (Xp22.3)	5 (7)	6 (5)								
Translocation	0 (0)	2 (1)								
Heart malformation (bicuspid and/or coarctation), n (%)	18 (24)	35 (28)								

^a^ *P* < 0.01 (unadjusted) between GH treated and untreated TS women at baseline and every follow-up (Student *t* test or Fisher exact test).

### HRQoL in TS: longitudinal analyses

HRQoL scores for women with TS at each 5 year-follow-up visit and for the reference population are shown in Table 3. Linear regression analyses of the association between the HRQoL scores and a selection of the tested somatic factors in TS are presented in Table 4. Older age was associated with lower HRQoL in the women with TS over time in most of the subscales used. However, when the women who had received GH in childhood were analyzed separately, HRQoL was not associated with age in any of the subscales (data not shown). The significant associations that are reported for the whole group in Table 4 did remain in the untreated group. Higher age at TS diagnosis was associated with lower HRQoL in all of the subscales used, except NHP Sleep.

**Table 3.** HRQoL in Women With TS at Baseline and 5 to 20 y Follow-Up and in Reference Population of Women From WHO-MONICA Study

	TS Baseline (n = 153)	TS 5-y Follow-Up (n = 102)	TS 10-y Follow-Up (n = 66)	TS 15-y Follow-Up (n = 43)	TS 20-y Follow-Up (n = 11)	Reference Population (n = 317)
Age, y, mean (SD); minimum–maximum	28.1 (10.9); 18–66	35.8 (11.3); 23–62	41.9 (11.1); 27–67	49.4 (11.5); 34–73	52.9 (11.7); 40–78	63.7 (9.0); 39–78
GH treatment (previous), n (%)	98 (64)	53 (52)	30 (45)	15 (34)	5 (46)	n.a.
PGWB						
Anxiety	22.9 (5.0)	22.9 (4.6)	23.7 (4.2)	23.4 (3.2)	22.6 (3.6)	23.9 (5.2)
Depressed mood	15.4 (2.9)	15.2 (2.8)^*a*^	15.6 (2.4)	15.3 (2.1)	15.9 (1.9)	15.8 (2.7)
Positive well-being	16.8 (3.3)	16.5 (3.2)	16.8 (3.1)	15.9 (3.2)	15.7 (2.5)	16.2 (3.8)
Self-control	14.8 (2.6)	14.7 (2.4)	15.0 (2.4)	14.5 (2.3)	15.3 (1.7)	15.4 (2.7)
General health	15.6 (2.5)	15.2 (2.8)	15.1 (2.9)	14.7 (2.6)	13.3 (2.4)	14.1 (3.2)
Vitality	17.1 (3.7)	16.5 (3.8)	16.8 (3.2)	16.7 (3.0)	17.0 (2.4)	17.1 (4.2)
Total score	102.5 (17.1)	101.0 (17.1)	103.1 (15.6)	100.6 (14.1)	101.3 (11.0)	102.6 (18.6)
NHP						
Energy	13.3 (26.9)	13.6 (25.0)	14.1 (28.8)	12.8 (25.6)	8.7 (20.6)	17.9 (31.7)
Emotional Reaction	11.5 (20.0)	13.8 (24.5)	8.9 (17.4)	9.2 (19.0)	1.5 (3.2)	10.2 (18.4)
Sleep	12.2 (21.7)	15.5 (24.0)	12.1 (18.7)	15.9 (25.5)	19.9 (28.6)	23.1 (27.7)
Pain	5.1 (17.0)	8.1 (20.9)	4.7 (12.9)	10.8 (21.7)	13.3 (26.9)	15.3 (26.1)
Physical Mobility	3.0 (8.3)	4.3 (11.6)^*a*^	4.5 (10.1)	7.9 (13.8)	15.6 (26.7)	9.3 (17.0)
Social Isolation	11.0 (22.1)	10.6 (22.5)^*a*^	6.9 (15.6)	12.2 (21.7)	13.8 (18.5)	5.9 (15.8)

Data are means (SD), unless otherwise specified. HRQoL instruments used: the PGWB and the NHP. UNIANOVA, age-adjusted model was used to compare HRQoL between each follow-up of the women with TS and the reference population.

Abbreviation: n.a., not applicable.

^a^ Statistically significant differences at the *P* < 0.01 level.

**Table 4.** Longitudinal Linear Regression Analyses of Associations Between HRQoL in TS and Somatic and Social Factors

	Age	GH Treatment^*a*^	Height^*a*^	Age at Diagnosis^*a*^	Use of Hearing Aid^*a*^
PGWB					
Anxiety	−0.03 (−0.08, 0.01)	0.15 (−1.57, 1.86)	0.04 (−0.07, 0.14)	−0.13 (−0.20, −0.06)^*b*^	−0.001 (−1.60, 1.60)
Depressed mood	−0.03 (−0.05, 0.001)	0.09 (−0.92, 1.10)	0.05 (−0.01, 0.11)	−0.08 (−0.11, −0.04)^*b*^	−0.31 (−1.25, 0.62)
Positive well-being	−0.05 (−0.08, −0.02)^*b*^	0.50 (−0.70, 1.70)	0.01 (−0.06, 0.08)	−0.05 (−0.10, −0.002)^*b*^	−0.02 (−1.15, 1.11)
Self-control	−0.01 (−0.04, 0.01)	0.14 (−0.81, 1.09)	0.02 (−0.04, 0.08)	−0.05 (−0.09, −0.01)^*b*^	−0.29 (−1.18, 0.60)
General health	−0.07 (−0.10, −0.05)^*b*^	−0.12 (−1.07, 0.83)	0.03 (−0.03, 0.09)	−0.04 (−0.08, −0.01)^*b*^	−0.95 (−1.83, −0.07)^*b*^
Vitality	−0.04 (−0.08, −0.01)^*b*^	1.29 (−0.02, 2.59)	−0.002 (−0.08, 0.08)	−0.08 (−0.13, −0.03)^*b*^	−0.11 (−1.34, 1.13)
Total score	−0.23 (−0.40, −0.07)^*b*^	2.15 (−3.96, 8.30)	0.14 (−0.23, 0.51)	−0.43 (−0.67, −0.20)^*b*^	−1.76 (−7.51, 4.00)
NHP					
Energy	0.03 (−0.24, 0.30)	−0.97 (−10.95, 9.01)	−0.25 (−0.85, 0.34)	0.54 (0.14, 0.95)^*b*^	0.69 (−8.76, 10.14)
Emotional reaction	0.04 (−0.17, 0.25)	−0.28 (−8.15, 7.60)	−0.57 (−1.03, −0.11)^*b*^	0.57 (0.26, 0.89)^*b*^	−0.92 (−8.37, 6.54)
Sleep	0.39 (0.17, 0.61)^*b*^	0.81 (−7.59, 9.20)	−0.25 (−0.75, 0.25)	0.19 (−0.15, 0.53)	−2.84 (−10.78, 5.10)
Pain	0.52 (0.34, 0.70)^*b*^,^*c*^	−0.12 (−6.83, 6.58)	0.12 (−0.28, 0.52)	0.29 (0.01, 0.56)^*b*^	4.53 (−1.67, 10.72)
Physical mobility	0.30 (0.19, 0.41)^*b*,*c*^	1.22 (−2.93, 5.37)	−0.07 (−0.32, 0.17)	0.21 (0.04, 0.37)^*b*^	4.77 (0.90, 8.66)^*b*^
Social isolation	0.09 (−0.12, 0.30)	1.86 (−6.20, 9.92)	−0.52 (−0.99, 0.04)	0.32 (−0.01, 0.65)	5.25 (−2.40, 12.89)
					

	Use of Levothyroxine^*a*^	Use of Analgesics^*a*^	Use of Antidepressant/Sedative^*a*^	Unemployed/Disability Pension^*a*^	Living Alone^*a*^
PGWB					
Anxiety	−0.05 (−1.31, 1.22)	−1.44 (−3.94, 1.07)^*c*^	−2.24 (−4.20, −0.28)^*b*,*c*^	−0.58 (−2.51, 1.35)	−0.01 (−1.25, 1.27)
Depressed mood	−0.21 (−0.54, 0.95)	−1.52 (−2.98, −0.06)^*b*,*c*^	−1.45 (−2.61, −0.31)^*b*,*c*^	−0.46 (−1.57, 0.65)	−0.05 (−0.79, 0.68)
Positive well-being	−0.25 (−0.65, 1.15)	−0.93 (−2.72, 0.86)^*c*^	−1.49 (−2.89, 0.09)^*c*^	−0.32 (−1.68, 1.03)	−0.66 (−1.53, 0.21)
Self-control	−0.14 (−0.55, 0.82)	−0.33 (−1.70, 1.03)^*c*^	−1.24 (−2.31, −0.17)^*b*,*c*^	−0.34 (−1.40, 0.72)	−0.15 (−0.84, 0.54)
General health	−0.12 (−0.59, 0.83)	−2.76 (−4.12, −1.39)^*b*,*c*^	−1.23 (−2.33, −0.12)^*b*,*c*^	−1.35 (−2.39, −0.31)^*b*^	−0.002 (−0.69, 0.70)
Vitality	−0.21 (−0.77, 1.21)	−1.44 (−3.41, 0.53)^*c*^	−2.01 (−3.55, −0.47)^*b*,*c*^	−0.19 (−1.29, 1.66)	0.21 (−0.75, 1.16)
Total score	−1.06 (−3.49, 5.62)	−8.52 (−17.44, 0.39)^*c*^	−9.77 (−16.75, −2.79)^*b*,*c*^	−2.79 (−9.64, 4.06)	−0.69 (−5.17, 3.79)
NHP					
Energy	−4.45 (−11.90, 2.99)	15.56 (0.14, 30.97)^*b*,*c*^	11.72 (−0.07, 23.51)^*c*^	−0.16 (−11.63, 11.32)	−0.36 (−7.70, 6.99)
Emotional Reaction	−2.21 (−8.12, 3.69)	17.71 (5.64, 29.79)^*b*,*c*^	9.22 (−0.11, 18.56)^*c*^	3.71 (−5.14, 12.55)	−0.37 (−6.17, 5.42)
Sleep	−1.72 (−8.23, 4.78)	17.81 (4.48, 31.13)^*b*,*c*^	11.57 (1.33, 21.80)^*b*,*c*^	4.34 (−5.26, 13.95)	1.99 (−4.19, 8.18)
Pain	0.85 (−4.35, 6.05)	32.34 (22.50, 42.18)^*b*,*c*^	4.44 (−3.82, 12.70)	7.53 (0.49, 14.57)^*b*^	−2.61 (−7.52, 2.31)
Physical Mobility	0.35 (−2.81, 3.52)	16.38 (10.18, 22.57)^*b*,*c*^	5.22 (0.22, 10.22)^*b*^	5.25 (1.26, 9.23)^*b*^	−0.16 (−2.90, 3.21)
Social Isolation	−3.44 (−9.34, 2.45)^*c*^	17.56 (5.46, 29.65)^*b*,*c*^	6.38 (−3.01, 15.77)^*c*^	−1.40 (−10.53, 7.74)	6.38 (0.49, 12.27)^*b*,*c*^

Values are coefficient estimates, *β* (99% CIs). HRQoL instruments used: the PGWB and the NHP.

^a^ Age-adjusted model.

^b^ Statistically significant.

^c^ Denotes scales in which a significant association was found in the reference population using UNIANOVA age-adjusted model. GH treatment, age at diagnosis, and employment status were not tested in reference population.

Lower HRQoL over time was found in the PGWB General Health and NHP Physical Mobility subscales in the women with TS who used a hearing aid (Table 4). No association was found between HRQoL and body mass index waist-hip ratio, number of external TS stigmata, karyotype, or presence of a congenital cardiovascular malformation (bicuspid aortic valve and/or coarctation of the aorta) (data not shown).

### Associations between HRQoL and comorbidity in women with TS vs reference population

There were no differences found in any subscale between the women with TS and the reference population of women after age adjustment at baseline, the 10-, 15-, and 20-year follow-ups (Table 3).

HRQoL was negatively correlated with age in the reference population (Table 4) in the NHP Pain (*β* = 0.49; 99% CI 0.07, 0.91) and Physical mobility (*β* = −0.49; 99% CI 0.23, 0.76) subscales. HRQoL was not associated with the use of a hearing aid or height in the reference population.

There was a negative association between HRQoL and the use of analgesics and medication for depression/anxiety in both patients and controls in most of the subsubscales (Table 4). The use of medication for hypertension and hyperlipidemia was not associated with HRQoL scores in TS or in the reference population (data not shown). Both groups reported more problems with social isolation if they were living alone (reference population: *β* = 6.18; 99% CI 1.44, 12.09).

## Discussion

This study evaluated HRQoL of adult women with TS with a focus on how GH treatment and comorbidity influence HRQoL during up to 20 years of follow-up and compared the HRQoL of women with TS to that of women in the general population from the same area. A large cohort of women with TS was followed in detail up to the age of 78, and HRQoL was measured using two validated, generic instruments at regular intervals.

### GH and height

No significant association between HRQoL and given GH treatment could be found in women with TS at baseline or during follow-up. This finding is similar to the other smaller studies that have compared GH-treated to untreated women with TS, in which no benefit of GH on HRQoL was found in younger women (11, 12).

HRQoL was only associated with adult height in the women with TS in one of the 13 subscales used (NHP Emotional reactions). The clinical relevance of this association is questionable because there were no significant associations found between the conceptually similar subscales in the PGWB (Anxiety and Depressed mood) and height (27). There was no association between HRQoL and height in the reference population. These results indicate that height is not a factor that affects HRQoL later in life in TS, which is in line with other large studies that reported no association between quality of life determinants and height, or height increase, after GH treatment (13, 28). However, three other studies have reported that height was positively correlated with the physical functioning domains of HRQoL (12, 14, 29). It is known from clinical experience that height is a main concern, especially for the younger women and girls with TS and/or their parents (30).

The introduction of GH treatment to treat short stature in TS in the late 1980s marked the beginning of a paradigm shift in the way women with TS are treated both in childhood and adulthood, which was not limited to GH treatment alone. Naturally, this created a cohort effect, or generational difference, within TS: those who had received modern treatment and care and those who had not.

A call for studies systematically evaluating the effects of GH treatment on quality of life was made by Cuttler and Rosenfield in 2011 (17). Because no randomized placebo-controlled trials of GH treatment in TS with long follow-ups have been published with HRQoL outcomes, this study has the best prerequisites to answer the question. Like Carel *et al.* (13) concluded in their study of HRQoL of younger women with TS in 2005, our findings call into question whether treating short stature in childhood with such a cumbersome and expensive treatment is justified when the height gain is relatively small. There is also a risk that the treatment contributes to “health care fatigue” in childhood that may cause the young women on the cusp of transition to adult care to abstain from further monitoring, which may put them at greater risk later in life unnecessarily.

### Age and age at diagnosis

TS could be considered a syndrome that entails early ageing and HRQoL decreased during the course of the follow-up, and there was a negative association between most of the HRQoL domains and age. HRQoL was also negatively associated with higher age at TS diagnosis, which confirms the idea that an early diagnosis and initiation of age-appropriate treatment is important for HRQoL later in life (31). Early diagnosis of TS makes appropriate medical, psychological, and educational interventions possible and also may increase the individual’s ability to cope with the disorder.

### Comorbidity in TS

Complications related to TS occurred at a relatively young age in both the GH-treated and untreated women, and more women with TS were on disability pensions as time progressed. This is all similar to the published literature (31–33).

Hearing loss was very common in TS and began early in life in contrast to the reference population. The use of a hearing aid was associated with worse HRQoL in the general health domain and physical mobility domain. Otological involvement has been shown to be associated with a lower self-esteem in TS in a large French cohort, but hearing loss was not associated with HRQoL in an Irish cohort of younger patients with TS (12, 13).

Genotype, body composition, cardiovascular malformations, and hypothyroidism were not associated with HRQoL, most of which is in line with several other studies (12, 13). This may be a result of the close and proactive treatment these women receive at the Turner Center because another study showed a strong association between HRQoL and cardiac involvement in younger women with TS (13).

### TS vs reference population

The similarity in HRQoL between women with TS and women in the population found in this study is in contrast to another study from Norway that found that the women with TS had lower overall life satisfaction, more difficulties with physical functioning, and lower self-perceived general health compared with age-matched controls at follow-up (33). However, response and follow-up rates were low in both the TS and control groups in that study, which affects generalizability and may explain the differences in results (29, 33).

Both women with TS and women in the population reported problems in the NHP Social isolation domain in relation to civil status. Because living alone was more common among women with TS, it may require special attention. It is not surprising that the use of analgesics and antidepressants was associated with lower HRQoL in both the population with TS and the reference population. Attention must also be given to ergonomically adapt the workplace environment, given the extra physical strain that short stature entails.

### Strengths and limitations

This study presents HRQoL results from a large cohort of genetically representative adult women with TS who have been carefully monitored over many years. However, according to the prevalence of TS (1 out of 2500 female births), only about half of the women with TS in the population have been diagnosed. It is possible that these undiagnosed women do not have considerable somatic, psychological, or social problems. This would, in turn, lead us to overestimate the problems that women with TS face and underestimate the influence of genetic makeup. However, this is true for all studies based on clinical TS cohorts. The karyotype distribution in this study was similar to other Turner registers (32, 34). The results may also have been skewed by the patients who were lost to follow-up, who died during the follow-up time, and who did not complete HRQoL questionnaires at their follow-up visits because the HRQoL results were analyzed using longitudinal statistical methods.

The comparison made between the population with TS and the reference population must be interpreted with caution because of the age disparity. The use of two instruments that have been validated in other chronic diseases increases the reliability of the results. Furthermore, women with TS and the women in the reference population scored similarly when assessing the association between HRQoL and use of analgesics and antidepressant medication. This suggests that the instruments are capable of measuring HRQoL in relation to medical problems.

## Conclusion

No association between HRQoL and GH treatment in childhood was found after age adjustment despite the average 5.7 cm taller final height in adult women with TS. HRQoL was not associated with genotype, body composition, hypothyroidism, or the presence of cardiovascular malformations (*i.e.*, the main stigmatas of TS) after up to 20 years of follow-up. However, HRQoL was compromised by age, age at diagnosis, hearing impairment, civil status, and employment, associations with some of which were also found in women without TS. Women with TS up to 78 years of age reported similar HRQoL to women recruited from the general population.

## Abbreviations:

HRQoL: health-related quality of life
MONICA: MONItoring of trends and determinants for CArdiovascular disease
NHP: Nottingham Health Profile
PGWB: Psychological General Well-Being index
TS: Turner syndrome
UNIANOVA: univariate ANOVA
WHO: World Health Organization
